# 

**Published:** 1904-03-26

**Authors:** 


					THE HOSIWIAL.
??- ' i
The Standard of highest purity.'?Lancet. March 2Gth, 1904.
terso^J.u ft* CADBURY'S
CADBUR Y& ?ygi
COCOA., E, JEH) COCOA
PERFECT FPOtnV X. ? NO DRUGS OR ALKALIES
H
6
3.
NO. 913. Vol. XXXV. [a WewspapCT.] SATURDAY,
March 26th, 1904.
^Subscription T?.erms,J pRICE THREEPENCE.

				

